# Meroterpenoid-Rich Fraction of the Ethanolic Extract from *Sargassum serratifolium* Suppressed Oxidative Stress Induced by *Tert*-Butyl Hydroperoxide in HepG2 Cells

**DOI:** 10.3390/md16100374

**Published:** 2018-10-09

**Authors:** Sujin Lim, Misung Kwon, Eun-Ji Joung, Taisun Shin, Chul-Woong Oh, Jae Sue Choi, Hyeung-Rak Kim

**Affiliations:** 1 Department of Food Science and Nutrition, Pukyong National University, 45, Yongso-Ro, Nam-Gu, Busan 48513, Korea; cocky0305@naver.com (S.L.); mskwon80@hanmail.net (M.K.); eunji2007@naver.com (E.-J.J.); choijs@pknu.ac.kr (J.S.C.); 2 Division of Food and Nutrition, Chonnam National University, 77, Yongbong-ro, Buk-gu, Gwangju 61186, Korea; shints@jnu.ac.kr; 3 Department of Marine Biology, Pukyong National University, 45, Yongso-Ro, Nam-Gu, Busan 48513, Korea; ohcw@pknu.ac.kr

**Keywords:** antioxidant, oxidative stress, *Sargassum serratifolium*, *tert*-butyl hydroperoxide, Nrf2

## Abstract

*Sargassum* species have been reported to be a source of phytochemicals, with a wide range of biological activities. In this study, we evaluated the hepatoprotective effect of a meroterpenoid-rich fraction of the ethanolic extract from *Sargassum* *serratifolium* (MES) against *tert*-butyl hydroperoxide (*t*-BHP)-treated HepG2 cells. Treatment with MES recovered the cell viability from the *t*-BHP-induced oxidative damage in a dose-dependent manner. It suppressed the reactive oxygen species production, lipid peroxidation, and glutathione depletion in the *t*-BHP-treated HepG2 cells. The activity of the antioxidants induced by *t*-BHP, including superoxide dismutase (SOD) and catalase, was reduced by the MES treatment. Moreover, it increased the nuclear translocation of nuclear factor erythroid 2-related factor 2, leading to the enhanced activity of glutathione S transferase, and the increased production of heme oxygenase-1 and NAD(P)H:quinine oxidoreductase 1 in *t*-BHP-treated HepG2 cells. These results demonstrate that the antioxidant activity of MES substituted the activity of the SOD and catalase, and induced the production of detoxifying enzymes, indicating that MES might be used as a hepatoprotectant against *t*-BHP-induced oxidative stress.

## 1. Introduction

Reactive oxygen species (ROS) are continuously generated in organisms as a by-product of aerobic respiration. They play a key physiological role in the stimulation of growth and immune response in living cells in response to intracellular and extracellular stimuli [1]. The ROS level is regulated by enzymatic and non-enzymatic antioxidant defense systems. Excessive ROS are instantly removed by intracellular antioxidant compounds, such as glutathione (GSH), vitamin C, and vitamin E, and are perpetually regulated by antioxidant enzymes, including superoxide dismutase (SOD), catalase, glutathione reductase, glutathione peroxidase, and glutathione *S*-transferase (GST) [2]. Reactive oxygen species damage major cellular biomolecules, such as DNA, protein, and lipids, leading to pathophysiological conditions, including diabetes mellitus, hypertension, obesity, dyslipidemia, cancer, and inflammation [3]. Thus, dietary supplementation of antioxidants has been used as a preventive or therapeutic strategy against oxidative damage caused by ROS.

The nuclear factor (erythroid-derived 2)-like 2 (Nrf2) is a basic region/leucine zipper transcription factor, which regulates the expression of antioxidants and detoxifying enzymes [heme oxygenase-1 (HO-1), NAD(P)H:quinone acceptor oxidoreductase 1 (NQO1), and GST]. Under basal conditions, Nrf2 is inactive in the cytosol by binding with Kelch-like ECH-associated protein 1 (Keap1), which inhibits the translocation of Nrf2 from the cytosol into the nucleus [4]. The dissociation of Nrf2 from its cytosolic repressor, Keap1, is a primary step in the activation of Nrf2, which induces the expression of phase II detoxifying enzymes [5]. NQO1, HO-1, and GST have been known to be target enzymes of Nrf2. NQO1 reduces quinone to hydroquinone by a detoxification reaction in biological systems [6]. HO-1 induces the enzymatic degradation of heme to produce carbon monoxide and biliverdin, which is subsequently converted into bilirubin, a strong antioxidant [7].

*Tert*-butyl hydroperoxide (*t*-BHP) is a well-known pro-oxidant, which induces oxidative damage in in vivo or in vitro models. The metabolic intermediates of *t*-BHP by cytochrome P-450 in hepatocytes generate toxic free radicals, such as peroxyl and alkoxyl radicals, resulting in oxidative damage to the cells [8]. Thus, *t*-BHP is widely used to induce oxidative damage in order to investigate the mechanism of hepatocyte injury [9]. HepG2 cells, a human hepatoma cell line, have been used to study the mechanisms of oxidative stress and xenobiotics metabolism, as they retain their endogenous antioxidants and xenobiotic-metabolizing enzymes [10].

Brown algae are a rich source of bioactive compounds, such as meroterpenoids, phlorotannins, fucoxanthin, sterols, and glycolipids [11,12]. *Sargassum serratifolium* (C. Agardh) is distributed throughout the coast of Korea and Japan. Recently, we found that the ethanolic extract of *S. serratifolium* exhibits a strong anti-inflammatory activity, and the active compounds involved were identified as sargahydroquinoic acid (SHQA), sargachromanol (SCM), and sargaquinoic acid (SQA) [13,14]. We obtained a meroterpenoid-rich fraction from the ethanolic extract of *S. serratifolium* (MES) by removing the salts and water-soluble saccharides. The combined amount of SHQA, SCM, and SQA in 100 g of MES was estimated to be 45.7 ± 2.2 g, indicating that MES contains a high amount of active components [15,16]. In this study, we investigated the hepatoprotective effect of MES on *t*-BHP-treated HepG2 cells, and confirmed that MES induced antioxidants and detoxifying enzymes by activating Nrf2. The findings in this study support that MES may be used as a source of nutraceuticals for hepatoprotection.

## 2. Results

### 2.1. Hepatoprotective Effect of MES

**Figure 1:**
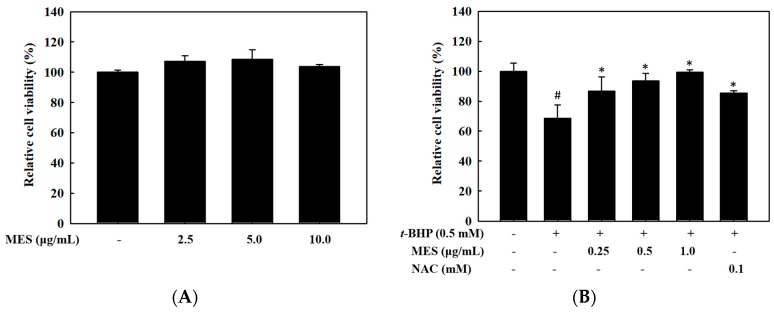
Hepatoprotective effect of MES on *t*-BHP-stimulated oxidative stress. (**A**) The cell viability of MES was measured by using Cell Proliferation Assay Kit. (**B**) The hepatoprotective effect was determined with various concentrations of MES in *t*-BHP-treated HepG2 cells. The data are the means ± SD of three independent experiments. # *p* < 0.05 indicates significant differences from the control group. * *p* < 0.05 indicates significant differences from the *t*-BHP treatment group.

To determine the non-toxic concentration range of MES in HepG2 cells, the MTS assay was performed with various concentrations of MES. As shown in Figure 1A, MES had no cytotoxicity at a concentration up to 10.0 μg/mL. The cells were treated with different concentrations of MES (0–1.0 μg/mL) in the presence of 0.5 mM *t*-BHP in order to evaluate the hepatoprotective effect of MES on *t*-BHP-treated cells. Treatment with *t*-BHP (0.5 mM) decreased the viability of HepG2 cells to 65%. However, treatment with MES recovered the cell viability in a dose-dependent manner (Figure 1B). Furthermore, treatment with 1.0 μg/mL MES completely recovered the viability of the cells damaged by *t*-BHP. N-acetyl-l-cysteine (NAC) (0.1 mM), as a positive control, showed an 80% of cell viability.

### 2.2. Effect of MES on the Inhibition of ROS Production and Lipid Peroxidation

**Figure 2:**
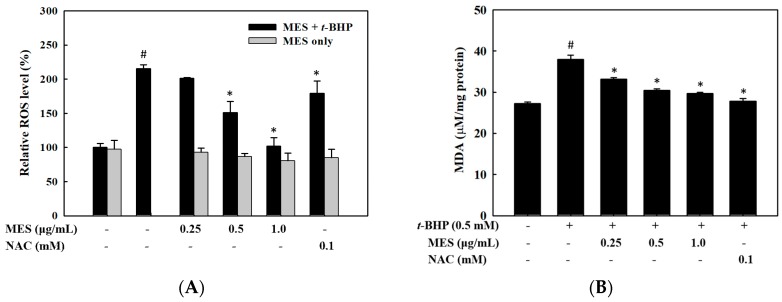

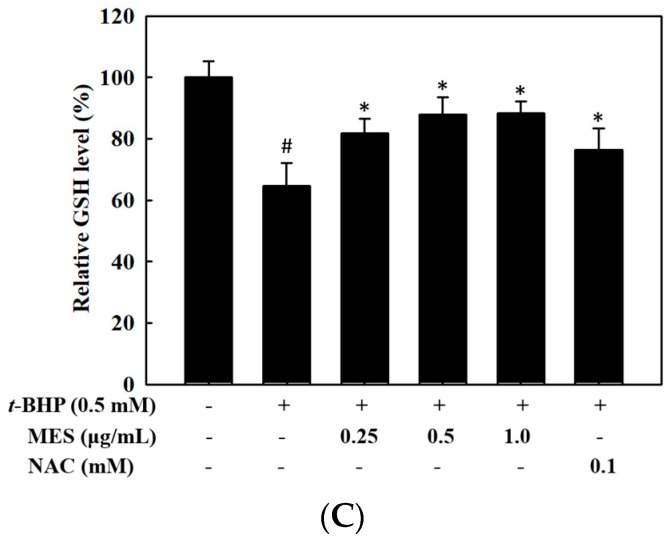
Inhibitory effect of MES on the reactive oxygen species generation, lipid peroxidation, and GSH depletion in *t*-BHP-treated HepG2 cells. HepG2 cells were treated with MES (0.25~1.0 μg/mL) and *t*-BHP. (**A**) ROS were measured by a fluorescent probe, DCFH-DA. (**B**) Lipid peroxidation was measured by using a TBARS assay. (**C**) The GSH level was measured by fluorescent probe, CMF-DA. The data are means ± SD of three independent experiments. # *p* < 0.05 indicates significant differences from the control group. * *p* < 0.05 indicates significant differences from the *t*-BHP treatment group.

We measured the intracellular ROS levels by using DCFH-DA to evaluate whether MES inhibits ROS production in *t*-BHP-treated HepG2 cells. As shown in Figure 2A, MES inhibited the *t*-BHP-induced ROS production in HepG2 cells in a dose-dependent manner. The ROS level in the 1.0 μg/mL MES treatment group was similar to that in the control group, which was significantly lower than that in the 0.1 mM NAC group. However, the reduction of the ROS level by MES-only treatment was negligible. To determine whether MES suppresses lipid peroxidation, we measured the intracellular MDA level with various concentrations of MES in the presence of 0.5 mM *t*-BHP. The increased MDA level by *t*-BHP treatment was reduced by MES treatment in a dose-dependent manner (Figure 2B).

### 2.3. Effect of MES on the Glutathione Levels

We measured the change of GSH levels using the fluorescent probe CMF-DA, as glutathione is a primary intracellular antioxidant molecule against oxidative stress. The decreased GSH level by *t*-BHP treatment was recovered by MES treatment in a dose-dependent manner (Figure 2C), indicating that MES may prevent the oxidation of GSH.

### 2.4. Effect of MES on SOD and Catalase Activities

**Figure 3:**
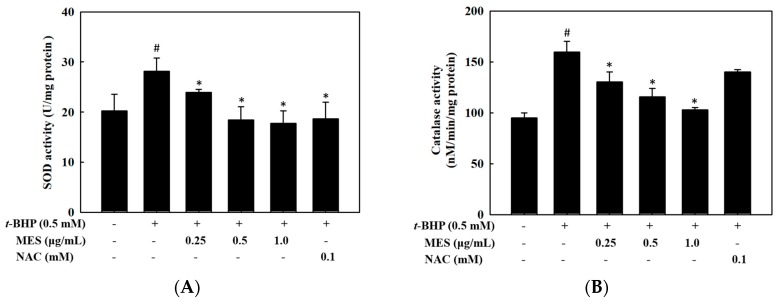
Effect of MES on antioxidant enzyme activities in *t*-BHP-treated HepG2 cells. Cells were treated with MES and *t*-BHP for 18 h. The cell lysates were prepared and used for superoxide dismutase (**A**) and catalase (**B**) activities. The values are the means ± SD of three independent experiments. # *p* < 0.05 indicates significant differences from the control group. * *p* < 0.05 indicates significant differences from the *t*-BHP treatment group.

The antioxidant enzymes, such as SOD and catalase, play a crucial role in the removal of the free radicals induced by oxidative damage. To determine the influence of MES on the antioxidant enzyme activities, we determined the activities of SOD (Figure 3A) and catalase (Figure 3B) after 24 h of treatment. As shown in Figure 3, the *t*-BHP treatment induced significantly increased activities of SOD and catalase (*p* < 0.05). However, the MES treatment reduced both the SOD and catalase activities in a dose-dependent manner, indicating an intrinsic antioxidant activity of the MES compensates both enzyme activities. The *N*-acetyl-l-cysteine (0.1 mM) did not recover the activity of SOD, whereas, it recovered the activity of the catalase.

### 2.5. Effect of MES on GST Activity and Expression of HO-1 and NQO1

**Figure 4:**
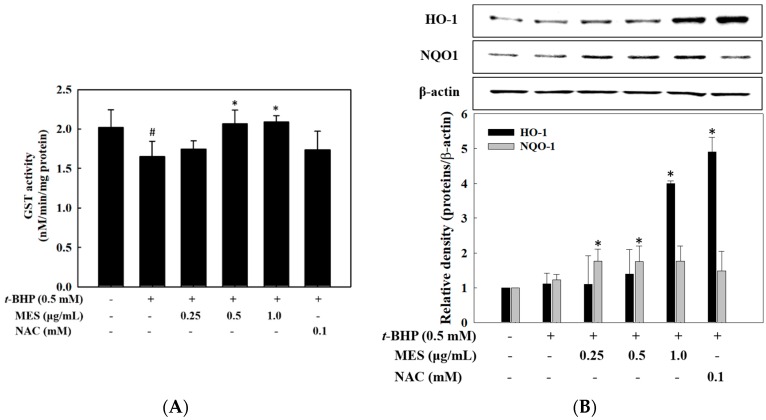
Effect of MES on the activity of GST and the productions of HO-1 and NQO1 in *t*-BHP-treated HepG2 cells. Cells were treated with MES and *t*-BHP for 18 h. (**A**) The GST activity was measured with a glutathione S-transferase assay kit. (**B**) The expressions of HO-1 and NQO1 were analyzed by Western blot. The data are means ± SD of three independent experiments. # *p* < 0.05 indicates significant differences from the control group. * *p* < 0.05 indicates significant differences from the *t*-BHP treatment group.

The activity of the detoxifying enzymes, such as GST, HO-1, and NQO1, was measured to confirm whether the detoxifying enzymes are involved in the increased viability of the *t*-BHP-treated HepG2 cells. The activity of the GST, reduced by *t*-BHP treatment, was recovered by treatment with MES in a dose-dependent manner (Figure 4A), however the expression of HO-1 and NQO1 was not affected by the *t*-BHP treatment. Particularly, the expression of HO-1 was increased by the highest concentration of the MES, while that of NQO1 was induced at a low concentration of MES (Figure 4B). The expression level of HO-1 by 1.0 μg/mL MES was enhanced four-fold compared with that in the control group. Moreover, 0.1 mM NAC (positive control) enhanced the expression of HO-1, but not the expression of NQO1.

### 2.6. Effect of MES on Nrf2 Expression and Nuclear Translocation

**Figure 5:**
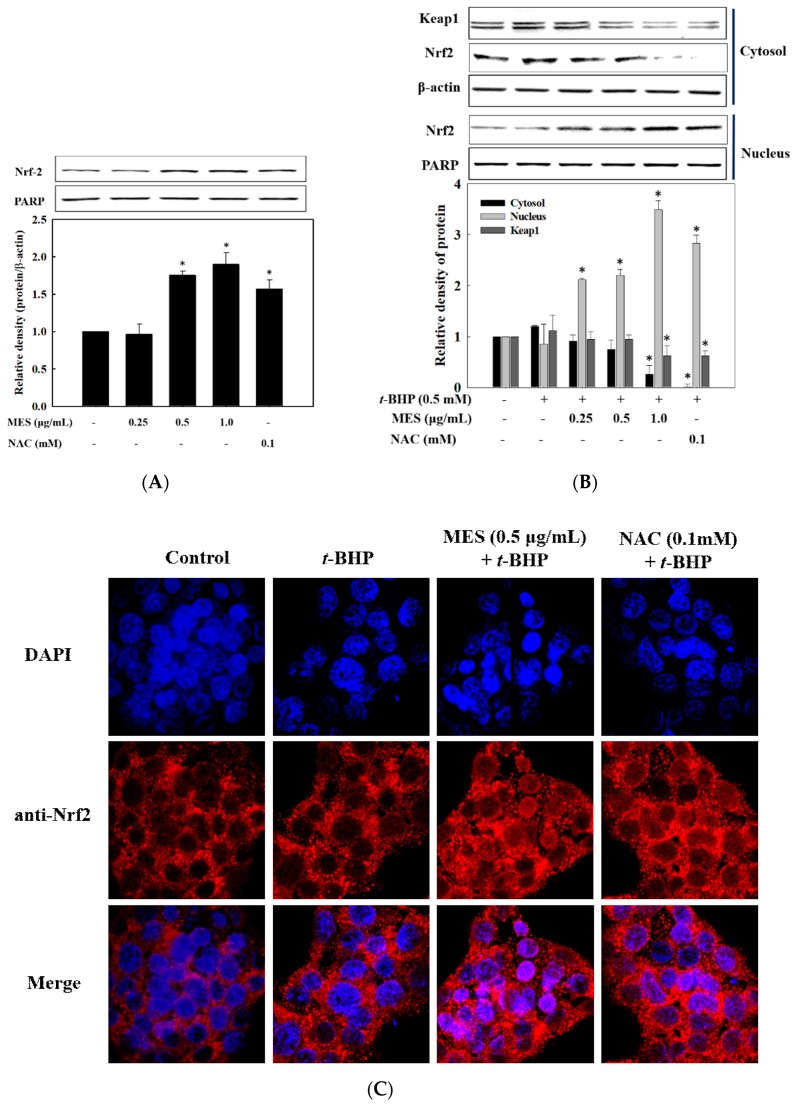
Effect of MES on the activation and translocation of Nrf2. (**A**) Cells were treated with indicated concentrations of MES for 1 h, and the nuclear fraction was analyzed with Western blot. (**B**) The cells were treated with MES and *t*-BHP for 1 h and separated cytosolic and nuclear fraction were analyzed with Western blot. (**C**) Cells were fixed and immunostained with anti-Nrf2 antibody (Alexa Fluor 586) and DAPI. The images were captured by confocal microscopy. The data are means ± SD of three independent experiments. * *p* < 0.05 indicates significant differences from the *t*-BHP treatment group.

As GST, HO-1, and NQO1 are regulated by the transcription factor Nrf2, we determined the translocation of Nrf2 into the nucleus after the treatment with MES. Cell lysates were separated into cytosolic and nuclear fractions, and the level of Nrf2 in each fraction was determined by Western blot. As shown in Figure 5A, the Nrf2 levels in the nuclear fraction were increased by MES treatment. To examine the effect of MES on the tranlocation of Nrf2 in the presence of *t*-BHP, the separated cytosolic and nucleic fractions were used to analyze the level of Nrf2. As shown in Figure 5B, the level of Nrf2 in the cytosol was significantly reduced in a dose-dependent manner (*p* < 0.05), whereas, the level of Nrf2 in the nuclear fraction increased. The increased level of Nrf2 by MES in the presence of *t*-BHP was higher than that by MES only. The level of Keap1 in the cytosol also decreased dose-dependently, inducing the Nrf2 translocation. Like MES, 0.1 mM NAC (positive control) enhanced the translocation of Nrf2 into the nucleus. We further confirmed the translocation of Nrf2 from the cytosol into the nucleus by confocal microscopy. In the control and *t*-BHP-treated cells, Nrf2 (red) was mostly observed in the cytosol. After stimulation by MES, Nrf2 was densely detected in the nucleus (Figure 5B). This result indicates that MES activates the transcriptional activity of Nrf2 via translocation into the nucleus in HepG2, suggesting that MES induces the expression of phase II detoxifying enzymes through the activation of Nrf2. 

### 2.7. Identification of Antioxidant Components in MES

To identify the antioxidant components in MES, we isolated SHQA, SCM, and SQA as described before [14]. The contents of SHQA, SCM, and SQA in 100 g of MES were estimated to be 37.6 ± 2.1, 6.23 ± 0.36, and 1.89 ± 0.10 g, respectively (Table 1). The inhibitory activity (IC_50_) of these components on the ROS production was determined using *t*-BHP-stimulated HepG2 cells. As shown in Table 1, the IC_50_ values of MES, SHQA, SCM, and SQA for the inhibition of ROS were 0.52 ± 0.08, 0.38 ± 0.04, 0.52 ± 0.12, and 0.35 ± 0.03 µg/mL, respectively.

## 3. Discussion

Oxidative damage is caused by an imbalance between the ROS level and antioxidant capacity in organisms. Recent studies have suggested that excess free radicals play a key role in the development and progression of liver injuries [17]. Thus, natural antioxidants have attracted attention for their safety, by preventing oxidative liver injuries, and the dietary consumption of antioxidants is recommended to prevent liver diseases [18]. Thus, we investigated the hepatoprotective effect of MES on *t*-BHP-treated HepG2 cells. The results showed that MES increased the viability of the *t*-BHP-treated cells by reducing the level of ROS and MDA and by increasing the level of GSH. Moreover, MES induced the expression of GST, HO-1, and NQO1 via the translocation of Nrf2 into the nucleus of the *t*-BHP-treated cells. The results also demonstrated that MES exhibits a hepatoprotective activity through its intrinsic antioxidant activity and by inducing detoxifying enzymes via the activation of Nrf2 in *t*-BHP-treated HepG2 cells.

*T*-BHP is a well-known oxidant that is used as a model compound to induce acute oxidative stress in vivo and in vitro [19]. It can be metabolized to free radical intermediates by cytochrome P450 in hepatocytes. The generated free radicals in the mitochondria are primarily eliminated by GSH, thus preventing damage to the lipids, proteins, and DNA [20]. Glutathione is a key antioxidant molecule against oxidative stress, and it can scavenge reactive radicals by conjugation and hydroperoxide reduction [21,22]. The reaction of ROS with the double bonds in polyunsaturated fatty acids produced lipid hydroperoxides, which form MDA by the degradation of peroxidized PUFAs. A high level of MDA caused by oxidative damages has been associated with various diseases, and it has been commonly been used as an index of lipid peroxidation [23]. The protective effect of MES on lipid peroxidation in HepG2 cells observed in the present study is consistent with the results of previous studies, which reported a similar effect by tea catechins [24], melanoidin [23], and quercetin [25] in the same cell line. These results showed that the MES-mediated protection against *t*-BHP-induced hepatotoxicity might be due to the intrinsic antioxidant activity of MES. 

Oxidative damage is one of the primary factors involved in the development of hepatopathy, including hepatitis and cirrhosis. HepG2 cells maintain an antioxidant defense system against the harmful effects of ROS [8]. In addition to the cellular antioxidant GSH, antioxidant enzymes SOD and catalase play a crucial role in the prevention of oxidative damage [26,27]. Superoxide dismutase can specifically catalyze the dismutation of superoxide radical anion to hydrogen peroxide and oxygen [28]. The resulting hydrogen peroxide is transformed to water and molecular oxygen by catalase. Thus, the alteration in the activity of antioxidant enzymes, such as SOD and catalase, might help protect against oxidative stress. In line with this, the tocotrienol-rich fraction from grape seeds suppressed the production of ROS and MDA [29]. Moreover, the fraction decreased the activity of the SOD and catalase in the *t*-BHP-treated HepG2 cells [29]. The decrease in the activity of SOD and catalase by MES in the *t*-BHP-treated HepG2 cells observed in the present study was presumed to be due to the reduced ROS and enhanced GSH levels. 

Several studies have shown that the hepatoprotective effect of natural compounds might be associated with the inhibition of oxidative stress by enhancing the antioxidant defense system and their intrinsic antioxidant activities [7,30]. Under normal conditions, Nrf2 sequestered by Keap1 is mostly distributed in the cytosol in an inactive form. Elevated levels of ROS by *t*-BHP treatment stimulates the Nrf2 activation, which regulates the cellular redox balance [7,31]. Activated Nrf2 binds to the antioxidant response element in the promoter regions of the antioxidants and detoxifying enzymes, including GST, HO-1, and NQO1 [32]. Glutathione S-transferase detoxifies the toxic substances by the enzymatic conjugation of reduced glutathione and xenobiotic substrates [26]. Heme oxygenase-1 catalyzes the degradation of heme to produce carbon monoxide and biliverdin, which is subsequently converted into bilirubin [7]. As a result, carbon monoxide and bilirubin act as strong antioxidants. NQO1 is an inducible protein under a variety of stress conditions, including oxidative stress, and plays multiple roles in cellular adaptation to oxidative stress [31]. Recent studies have reported that GST, HO-1, and NQO1 are induced by various phytochemicals via the activation of Nrf2, to combat oxidative damage [27,30]. The results of the present study showed that Nrf2 is translocated into the nucleus, upregulating the downstream detoxifying enzymes including NQO1, HO-1, and GST, evidenced by fluorescence assay and western blotting. This suggests that MES protects oxidative damage by stimulating the production of antioxidants and by detoxifying enzymes via Nrf2 activation and its intrinsic antioxidant activity in the *t*-BHP-treated HepG2 cells.

In our previous paper, we found that MES prevented diet-induced obesity and hepatic steatosis [15]. The major components for inhibiting the triglyceride (TG) accumulation in adipocytes were identified as SHQA, SCM, and SQA, which showed two or three times lower IC_50_ values than MES. However, the inhibitory effect of MES on diet-induced TG accumulation in hepatocytes was mainly caused by SCM and SQA, but SHQA did not potently contribute to the inhibition of TG accumulation in hepatocytes. Thus, the inhibition of the lipid accumulation in adipocytes was principally caused by SHQA, SCM, and SQA, however, that in the hepatocytes was caused by SCM, SQA, and unidentified components in MES. Additionally, MES inhibited the expression of intracellular adhesion molecule 1 (ICAM-1) and vascular cell adhesion molecule 1 (VCAM-1) in high cholesterol died (HCD)-fed mice [16]. SHQA showed an 8.8-fold higher inhibitory activity on the expression of VCAM-1 than MES, however, it was not effective in the inhibition of the ICAM-1 expression in TNF-α-treated HUVECs, suggesting selective effects of VCAM-1 [16]. This study investigates the antioxidant activity of MES and evaluates its hepatoprotective effect on *t*-BHP-treated HepG2 cells, as MES contains high levels of SHQA, SCMs and SQA. In this study, the IC_50_ value of MES and SCM for scavenging ROS radicals was similar, whereas, that of SHQA and SQA were less than MES. Based on the IC_50_ values and the content of the three compounds, SHQA is the main antioxidant component in MES. Also, MES may contain a marginal antioxidant activity, which would be caused by unidentified compounds. Although SHQA and SQA showed high antioxidant activity compared with MES, MES has an advantage for the commercial utilization of an antioxidant agent, because the isolation of each component requires a high cost and complicated procedure. This is an initiating study to assess the potency of *S. serratifolium* for hepatoprotection. MES showed cytoprotective properties by regulating the biochemical/molecular markers related to oxidative stress in the *t*-BHP treated cells, important pharmacological properties are largely uncentain. Further animal studies are required to identify the molecular target(s) of MES and its active components.

## 4. Materials and Methods

### 4.1. Materials

HepG2 cell line and Eagle’s minimum essential medium (EMEM) were purchased from ATCC (Manassas, VA, USA). Fetal bovine serum (FBS) and 0.25% trypsin–EDTA were purchased from Gibco-BRL Life Technologies (Grand Island, NJ, USA). The CellTiter96 Aqueous One Solution Cell Proliferation Assay kit was obtained from Promega (Madison, WI, USA). 5-Chloromethylfluorescein diacetate (CMF-DA), 4′6-diamidino-2-phenylindole (DAPI), protein marker, enhanced chemiluminescence (ECL), NE-PER Nuclear and Cytoplasmic Extraction Reagents, and the BCA protein assay kit were obtained from Thermo (Waltham, MA, USA). *Tert*-butyl hydroperoxide (*t*-BHP), 2,7-dichlorofluorescein diacetate (DCFH-DA), dimethyl sulfoxide (DMSO), and N-acetyl-cysteine (NAC) were purchased from Sigma-Aldrich (St. Louis, MO, USA). The antibodies against HO-1 (ab13248), NQO1 (ab34173), and anti-Nrf2 antibody (ab206893) were obtained from Abcam (Cambridge, UK). β-Actin (sc-47778), PARP (sc-7150), Nrf2 (sc-365949), and horseradish peroxidase-conjugated secondary antibodies (sc-2031) were purchased from Santa Cruz Biotechnology (Santa Cruz, CA, USA). The superoxide dismutase (SOD), catalase, and glutathione S-transferase (GST) assay kit were purchased from Cayman Chemical Co. (Ann Arbor, MI, USA). 

### 4.2. Preparation of MES and Isolation of Chemical Components

*S. serratifolium* was collected along the coast of Busan, South Korea, in May 2017. The specimen identity was confirmed by an algal taxonomist (C.G. Choi) at the Department of Ecological Engineering, Pukyong National University, Republic of Korea. The collected sample was air-dried and ground. One and half kilograms of the dried sample were extracted twice, with 70% ethanol (6 L each time) at 70 °C for 3 h. The combined extract was filtered with an ultrafiltration unit (MWCO, 50 kDa) and was concentrated until a lipophilic fraction was separated from the salt water. The lipophilic fraction was concentrated by a rotary vacuum evaporator (Eyela N3010, Tokyo, Japan) at 45 °C until the water content was less than 5.5%, and was used for this study. From the 1.5 kg of dried sample, 120 g of the MES was obtained. The isolation and quantification of SHQA, SCM, and SQA were performed according to the method described previously [14,33]. 

### 4.3. Cell Culture and Viability Assay

The HepG2 cells (ATCC, Manassas, VA, USA) were cultured in EMEM media containing 10% FBS in a humidified atmosphere of 5% CO_2_. The HepG2 cells were plated in a 96-well microplate (4 × 10^4^ cells/well) and incubated for 24 h. The culture media were replaced by 100 μL of MES (2.5, 5.0 and 10.0 μg/mL), diluted with a culture medium, and then incubated for 24 h. The cell viability was measured by CellTiter96 Aqueous One Solution Cell Proliferation Assay kit, according to manufacturer’s instructions. After 1 h of incubation at 37 °C, the plate was measured with a microplate reader (GloMax-multi detection system, Promega, Madison, WI, USA) at 490 nm. The MES stock solution (100 ug/mL) was made by dissolving in DMSO, and the working solution was made with a culture medium by diluting with a culture medium to obtain appropriate concentration.

### 4.4. Determination of ROS Production

Intracellular ROS level was determined by the oxidant-sensitive fluorescent probe DCFH-DA, as described previously [27]. The HepG2 cells were plated in a 96-well microplate (4 × 10^4^ cells/well), and then incubated for 24 h. The cells were treated with MES (0.25, 0.5, and 1.0 μg/mL) diluted with a culture medium, or MES and 0.5 mM of *t*-BHP, and then incubated for 1 h. The media was changed with 20 μM of DCFH-DA solution and incubated at 37 °C for 30 min. The fluorescence intensity was measured at an excitation wavelength of 485 nm and the emission wavelength of 523 nm, using amicroplate reader.

### 4.5. Measurement of Lipid Peroxidation

The lipid peroxidation was determined with a malondialdehyde (MDA) concentration using a thiobarbituric acid reactive substance (TBARS) assay kit from Cayman Chemical Company (Ann Arbor, MI, USA). Briely, the HepG2 cells were plated in six-well plates (1.2 × 10^6^ cells/well), and were then treated with an indicated concentration of MES and 0.5 mM *t*-BHP for 24 h to give enough exposing time for the MES. After treatment, the cells were scraped off and suspended in PBS. The suspended cells were sonicated and measured the concentration of MDA according to the manufacturer’s instructions. The absorbance of the product was measured at 540 nm using a microplate reader. The result was expressed as micromoles of MDA equivalents TBARS per microgram of protein.

### 4.6. Determination of Glutathione Level

The intracellular glutathione concentration was determined using the fluorescent probe CMF-DA, described in our previous research [27]. The HepG2 cells were plated in 96-well microplates (5 × 10^4^ cells/well), and then incubated for 24 h. After incubation, the media were changed with MES (0.25, 0.5, and 1.0 μg/mL), diluted with a culture medium, and 0.5 mM of *t*-BHP for 1 h. The media was changed with 10 μM of CMF-DA solution, and incubated at 37 °C for 1 h. The fluorescence intensity was measured at an excitation wavelength of 485 nm and the emission wavelength of 523 nm, using microplate reader.

### 4.7. Measurement of Antioxidant Enzyme Activities

The HepG2 cells cultured in six-well plates (1.2 × 10^6^ cells/well) were treated with the indicated concentration of MES and 0.5 mM of *t*-BHP for 18 h, since the change of the antioxidant enzyme activities were detectable after 18 h of MES treatment. After incubation, the cells were washed and harvested with a rubber scraper. The cells were suspended in PBS and sonicated for 10 s on ice. The cell lysates were centrifuged at 10,000× *g* for 15 min. The supernatants were stored at −80 °C, until required by the experiments. *N*-acetyl–cysteine (NAC) was used as a positive control. The catalase, SOD, and GST activities were measured using the Cayman Assay kit, according to the manufacturer’s instructions. 

### 4.8. Separation of Nuclear and Cytosolic Extract

The HepG2 cells (1.2 ×10^6^ cell/well) were treated with indicated concentrations MES and 0.5 mM of *t*-BHP for 1 h. The cells were washed with ice-cold PBS. The harvested cells were centrifuged at 1000× *g* for 5 min at 4 °C. The nuclear and cytosolic fractions were separated using NE-PER Nuclear and Cytoplasmic Extraction Reagent (Thermo, Waltham, MA, USA), according to our previous paper [30]. The separated fractions were stored at −70 °C, until further use.

### 4.9. Western Blotting

The HepG2 cells cultured in six-well culture plates (1.2 × 10^6^ cells/well) were treated with the indicated concentrations of MES and 0.5 mM of *t*-BHP for 18 h. The cells were lysed with a lysis buffer (50 mM Tris-HCl, pH 7.5, 150 mM NaCl, 1% NP-40, 1% Tween-20, 0.1% SDS, 1 mM Na_3_VO_4_, 10 μg/mL leupeptin, 50 mM NaF, and 1 mM PMSF) on ice for 30 min. After incubation, the cell lysates were centrifuged at 12,000× *g* for 20 min, the supernatants were transferred and determined the protein concentration using a BCA protein assay kit (Thermo, Waltham, MA, USA). Aliquots of proteins (40 μg) were separated by SDS-PAGE and were transferred onto a nitrocellulose membrane (Millipore, Burlington, MA, USA). The membrane was incubated with a primary antibody for 2 h. After washing with TBST, the membrane was treated with horseradish peroxidase-conjugated secondary antibody for 1 h. The proteins were detected using an ECL detection reagent. A densitometric analysis of the data was performed using a cooled CCD camera system EZ-Capture II and CS analyzer version 3.00 software (ATTO Co., Tokyo, Japan).

### 4.10. Immunofluorescence Analysis

To determine the nuclear localization of Nrf2 in the HepG2 cells, the cells were cultured on eight-well chamber slides (SPL Life Sciences Co., Gyeonggi-do, Korea) for 24 h, and were treated with 0.5 μg/mL of MES and 0.5 mM of *t*-BHP for 1 h at 37 °C. The treated cells were fixed in 4.0% (*w*/*v*) paraformaldehyde for 15 min at room temperature, and then permeabilized with 0.5% (*v*/*v*) Triton X-100 in PBS for 10 min. The permeabilized cells were blocked with 3% BSA in PBS for 1 h. The cells were incubated overnight in an anti-Nrf2 antibody (Alexa Fluor 568, Abcam, Cambridge, UK) at 4 °C. Then, the nuclei were stained with 2 μg/mL DAPI and observed using a LSM700 Laser scanning confocal microscope (Carl Zeiss, Oberkochen, Germany).

### 4.11. Statistical Analysis

All of the data were analyzed as the mean ± standard deviation (SD) of three independent experiments, unless otherwise indicated. The data analysis was performed using ANOVA, followed by the Bonferroni test. *p* < 0.05 were considered as statistically significant. SPSS for Windows, version 10.07 (SPAA Inc., Chicago, IL, USA), was used for the analyses. 

## 5. Conclusions

We have demonstrated that MES suppresses the production of ROS and MDA, and increases the cellular level of GSH in *t*-BHP-treated HepG2 cells. It also induces the production of GST, HO-1, and NQO1. Although MES showed preferential properties by regulating the antioxidant markers related to oxidative stress, important pharmacological profiles are largely unknown. Specifically, it is important to identify the molecular target(s) of MES and its active components. The active compounds have different ROS scavenging activities in the *t*-BHP-treated cells; however, the individual targets for antioxidation are uncertain. Hence, it will be critical to further investigate molecular and biochemical targets in order to better understand the pharmacological and toxicological profiles of the active components

## Figures and Tables

**Table 1:** Composition and inhibitory activities of MES and isolated components on reactive oxygen species (ROS) in *tert*-butyl hydroperoxide (*t*-BHP)-stimulated HepG2 cells.

Compounds	IC_50_^a^ (µg/mL)	Composition (%) ^b^
MES	0.52 ± 0.08	100
Sargahydroquinoic acid	0.38 ± 0.04	37.6 ± 2.10
Sargachromenol	0.52 ± 0.12	6.23 ± 0.36
Sargaquinoic acid	0.35 ± 0.03	1.89 ± 0.10

^a^ IC_50_ was measured with inhibition of ROS production by DCFH-DA fluorescence probe in *t*-BHP-induced HepG2 cells. ^b^ Composition shows amounts of active compounds in 100 g of MES.
